# Identifying the best FDA-endorsed healthy label designs through best-worst scaling experiment

**DOI:** 10.1017/S1368980025100542

**Published:** 2025-06-16

**Authors:** Jianhui Liu, Bachir Kassas, John Lai, Rodolfo M. Nayga

**Affiliations:** 1 University of Florida, Gainesville, FL, USA; 2 Texas A&M University, College Station, TX, USA; 3 Korea University, Seoul, South Korea

**Keywords:** Best-worst scaling, Front-of-pack labels, Food labelling policy, Food and Drug Administration

## Abstract

**Objective:**

This study aims to assess consumer preferences for fifteen proposed front-of-package ‘Healthy’ label candidates under the Food and Drug Administration’s (FDA) of the US updated guidelines for the ‘Healthy’ label. The goal of this study is to identify which label designs best align with consumer preferences, thereby supporting the FDA’s efforts to promote healthier dietary choices through effective labelling.

**Design:**

A best-worst scaling (BWS) experiment was conducted using a balanced incomplete block design to assess consumer preferences for the fifteen FDA-proposed ‘Healthy’ labels. Participants completed fifteen best-worst scaling choice tasks where they identified the ‘best’ and ‘worst’ design from three randomly presented options in each task.

**Setting:**

The experiment was conducted in a controlled laboratory setting in the USA.

**Participants:**

Three hundred and eight US adult consumers who are primary household shoppers without dietary restrictions.

**Results:**

Results from the random parameter logit model indicate that labels 12 and 8 emerged as the most preferred designs, with preference shares of 16·7 and 16·1 %, respectively. These two labels featured a prominent ‘Healthy’ display with bold blue font, balanced colour themes and check marks, which likely contributed to their appeal. The Krinsky and Robb bootstrapping method confirmed the statistical significance of the preferences for these labels over others.

**Conclusions:**

This study identifies two labels as the most preferred FDA-proposed ‘Healthy’ label designs, offering clear guidance to policymakers on effective labelling strategies. By adopting a consumer-preferred design, the FDA’s ‘Healthy’ label may have greater potential to influence healthier food choices.

On 29 September 2022, the Food and Drug Administration (FDA) of the USA published a proposal to update the definition of the term ‘Healthy’, which can be voluntarily used on food products as a front-of-package (FOP) label^(1)^. This proposal marks a significant shift from the previous definition established in 1994 and aims to align labelling practices with current nutrition science and the Dietary Guidelines for Americans^(1)^. Under the proposed rule, products would qualify as ‘Healthy’ by meeting updated criteria that emphasise limits on added sugars, sodium and saturated fat. The regulatory process was informed by several prior actions, including a public meeting in March 2017 and earlier guidance issued in 2016 to clarify the use of the term. His regulatory change is likely and expected to have broad implications, as FOP labelling policies have been shown to prompt product reformulation by manufacturers, shape dietary guidance provided by health professionals and influence consumer purchasing and eating behaviours^(1,2)^. In addition to the updated definition, the FDA has been exploring the development of a standardised ‘Healthy’ symbol. In 2021 and 2022, the FDA has proposed fifteen potential FOP labels (see Fig. 1) to signal food products that comply with the new regulation for ‘Healthy’, but it is expected to make a final determination on one label for implementation. While a final determination has yet to be made, consumers’ preferences and perspectives on these label prototypes are still unclear, yet they can play a crucial role in determining the overall effectiveness of FOP labels in meeting the FDA’s goal of nudging consumers toward healthier food choices^(3–5)^. Our study employs a best-worst scaling (BWS) (MaxDiff) experiment to measure consumer preferences for the candidate labels, allowing us to assess the relative ranking of the labels based on respondents’ choices. In doing so, this study aims to provide direct and actionable insights for policymakers in selecting a label that is most preferred by consumers.

**Figure 1.**
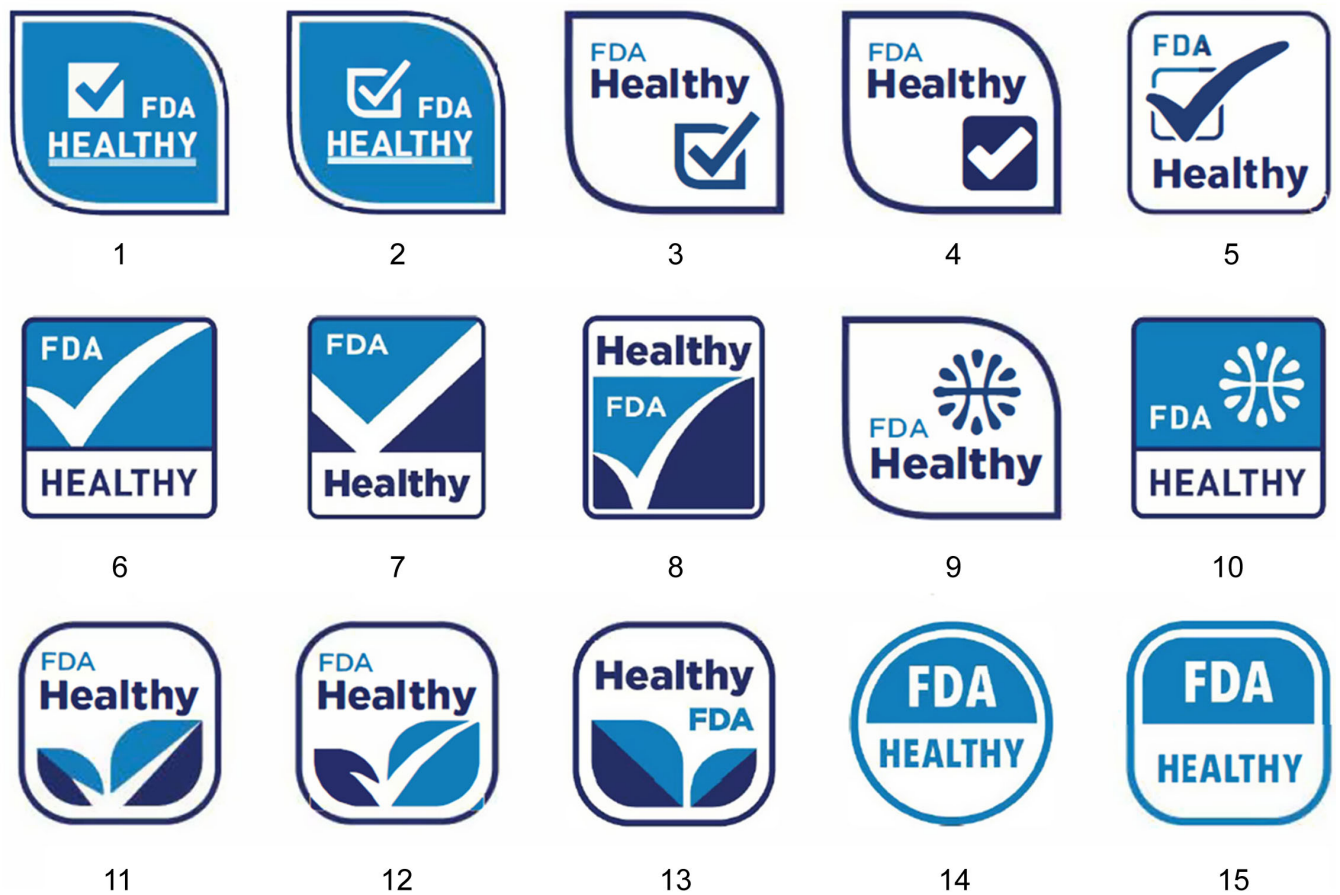
Candidates of proposed FDA-endorsed healthy labels.

## Experimental design

A laboratory experiment was conducted in the Southern region of the USA in April 2024. The experimental protocol was approved by the Institutional Review Board of a large Land Grant University and was pre-registered with the American Economic Association’s registry. In March 2024, subjects were recruited using online platforms (e.g. Facebook community groups); invitations via university listservs that contain students, faculty and staff; and banners placed in various locations (e.g. Publix, Walmart, Whole Foods and Target) around town. Interested participants were required to complete a short prescreening survey to determine their eligibility. Eligible participants were US citizens or residents, 18 years or older, primary household shoppers and without dietary restrictions. Among 1999 screened individuals, 531 met the eligibility criteria and were invited to participate in the experiment. Of those invited, 308 scheduled an appointment and completed the study session. The remaining eligible individuals did not participate.

**Figure 2.**
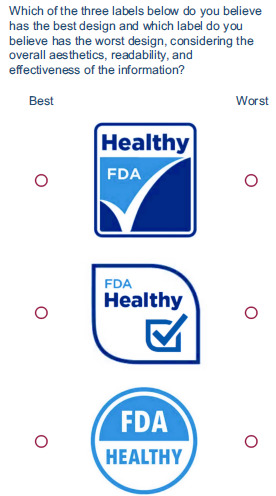
Sample choice task.

The BWS experimental methodology was used to measure consumers’ preferences for the fifteen candidate ‘Healthy’ FOP labels proposed by the FDA. The BWS is a discrete choice modelling technique that has an advantage in preference elicitation and estimations of relative importance or utility^(6,7)^. A balanced incomplete block design^(8)^ was used in RStudio to determine the allocation of the fifteen labels across choice tasks. This design is the most widely used in BWS experiments due to its balanced and orthogonal properties, which ensure that each choice (i.e. label) appears an equal number of times^(8)^. The balanced incomplete block design resulted in fifteen BWS choice tasks (blocks), each containing three options (alternatives). To avoid ordering effects, both the order of the choice tasks and the choices within each choice task were randomised across subjects. Subjects were provided with detailed experimental instructions before being asked to carefully evaluate each label, focusing on aesthetics, readability and the effectiveness of information presentation, as these are highly important factors that can determine the effectiveness of FOP labels^(9,10)^. In each choice task, participants were asked to select the ‘best design’ and the ‘worst design’ among the three choices. Figure 2 illustrates a sample BWS choice task. The data were collected via Qualtrics. A completed STROBE checklist is provided as supplementary material to support the transparent reporting of this study.

## Results and discussion

Table 1 presents the demographic characteristics of our sample. We found that 68·5 % of respondents in our study were female, aligning with prior research indicating that women are predominantly the primary grocery shoppers for households^(11)^. Two primary methods, counting and modelling, are used for analysing BWS experimental data^(12,13)^. We adopt the modelling approach, which statistically tests the differences between preferences for the various labels.^1^ A random parameter logit model was used to estimate consumer preferences, which is presented in Table 2. The coefficients indicate the relative preference for each of the fourteen labels, with Label 15 serving as the baseline and therefore omitted from the model. The significance of the sd confirms the existence of heterogeneity in consumer preferences for the ‘Healthy’ labels. The results indicate that Label 12 and Label 8 are the most preferred designs among participants. These two labels feature a heavy emphasis on the ‘Healthy’ wording, which is displayed prominently in bold contrasting blue font. Additionally, the balanced colour themes in these labels, featuring combinations of white, light blue and navy blue, along with the inclusion of check marks, likely contribute to their preference among consumers.

**Table 1.** Summary statistics of demographic variables

Variable	Category	Percentage (%)
Gender	Male	31·5
	Female	68·5
Age	18–24 years	60·1
	25–34 years	27·3
	35–44 years	5·8
	45–54 years	3·6
	55–64 years	1·3
	65+ years	1·9
Education	Less than High School	0·0
	High School Graduate	32·1
	Some College, No Degree	11·7
	Associate’s Degree	23·1
	Bachelor’s Degree	27·9
	Graduate, Doctoral or Professional Degree	27·9
Income	Less than $10 000	12·3
	$10 000–$14 999	6·8
	$15 000–$24 999	16·6
	$25 000–$34 999	17·9
	$35 000–$49 999	9·4
	$50 000–$74 999	11·7
	$75 000–$99 999	5·8
	$100 000–$149 999	10·7
	$150 000–$199 999	6·2
	$200 000+	2·6
Race	White	61·4
	Black or African American	5·2
	Asian	20·1
Ethnicity	Hispanic	24·4

**Table 2.** Random parameter logit (RPL) model estimations, preference shares and CI

Label	Coefficient	Std. error	Preference share (%)	95 % CI
Mean of estimates				
Label 01	−0·269***	0·089	2·4	2·0, 2·7
Label 02	0·168*	0·094	3·7	3·1, 4·2
Label 03	1·179***	0·088	10·0	8·7, 11·5
Label 04	0·981***	0·090	8·3	7·2, 9·5
Label 05	0·879***	0·104	7·5	6·4, 8·8
Label 06	1·073***	0·075	9·1	8·0, 10·2
Label 07	0·356***	0·088	4·4	3·8, 5·1
Label 08	1·642***	0·121	16·1	13·2, 19·1
Label 09	−0·772***	0·099	1·4	1·2, 1·7
Label 10	−0·761***	0·089	1·5	1·2, 1·7
Label 11	1·049***	0·104	8·9	7·5, 10·3
Label 12	1·681***	0·110	16·7	14·2, 19·4
Label 13	0·399***	0·106	4·6	3·8, 5·5
Label 14	−0·222	0·159	2·5	1·8, 3·3
Label 15	omitted	–	3·1	2·8, 3·5
Sd of estimates				
Label 01	0·752***	0·102		
Label 02	0·903***	0·103		
Label 03	0·672***	0·113		
Label 04	0·726***	0·101		
Label 05	1·284***	0·107		
Label 06	0·288*	0·161		
Label 07	1·031***	0·105		
Label 08	1·460***	0·123		
Label 09	0·764***	0·113		
Label 10	0·770***	0·094		
Label 11	1·152***	0·115		
Label 12	1·279***	0·116		
Label 13	1·383***	0·118		
Label 14	2·325***	0·184		
Label 15	omitted	–		

Notes: Label 15 was selected as the baseline and thus omitted in the RPL model. Significance levels: ***P < 0·01, **P < 0·05, *P < 0·1.

**Figure 3.**
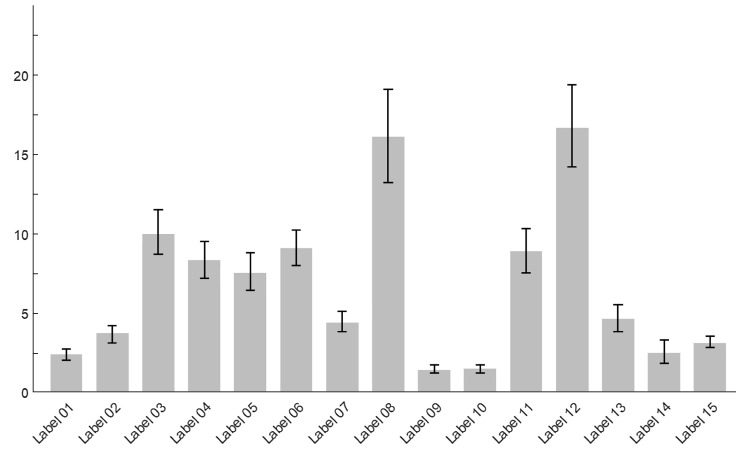
Mean preference shares and CI.

Preference shares, which represent the proportion of times each label is selected as the most or least preferred relative to others, are calculated for each label using the Krinsky and Robb bootstrapping method^(14)^. Specifically, we drew 1000 preference shares for each label from multivariate normal distributions based on the random parameter logit model. Columns 4 and 5 of Table 2 summarise the preference shares and their corresponding 95 % CI for each label. Figure 3 graphically illustrates the mean preferences for each label. Notably, Label 12 and Label 8 were identified as the best designs by 16·7 and 16·1 % of participants, respectively. The 95 % CI generated from the Krinsky and Robb method also suggest that Label 12 and Label 8 are statistically preferred over all other options in terms of aesthetics, readability and the effectiveness of information presentation. While Label 12 has the highest preference share, it is not statistically different from the preference share of Label 8.

### Concluding remarks and policy implications

This study uses the BWS experiment in a laboratory setting to elicit consumers’ preferences for fifteen proposed FDA-endorsed Healthy labels. Estimates from the random parameter logit model and Krinsky Robb method suggest that Label 12 and Label 8 were identified as the ‘best designs’, particularly in terms of aesthetics, readability and effectiveness in information presentation. The findings of this study offer preliminary insights that may help inform future decisions by the FDA and policymakers. The results provide insights into consumer preferences for different label designs, potentially enabling the FDA to select a label that resonates most with consumers. Food labels often fail to influence consumer behaviour when they do not align with consumer preferences or are poorly received^(3,15–17)^. Labels that are visually appealing, easy to read and effectively convey information are more likely to impact purchasing decisions^(9,10)^. By identifying the label designs most favoured by subjects, this study contributes to knowledge about which of the fifteen candidates’ FDA labels will likely be most effective in capturing consumers’ attention in terms of aesthetics, readability and the effectiveness of information presentation. This contribution is important in the food market, where many distractions can lead consumers to choose less healthy foods and move away from following the American dietary guidelines. Further, our study offers valuable guidance for future labelling policy investigation, as the methodology and results may serve as a model for evaluating and implementing other types of FOP labels.

### Limitations and directions for future studies

Although this study provides valuable guidance for policymakers and has the potential to offer insights for future labelling policy investigations, a few limitations must be acknowledged. First, although our study was able to identify the top candidate labels among the consumers, the influence of these labels remains unclear. Future research should test which of these top candidate labels would be most effective in inducing consumers to choose healthier food products. Furthermore, given that consumer preferences are influenced by numerous factors^(18,19)^ – and the drivers of preference in our study remain unclear – future research could explore the underlying factors shaping consumer responses to these label designs. Additionally, while our recruitment approach allowed access to a broad audience, it may have introduced selection bias by overrepresenting individuals who are more engaged in online communities or academic settings. The final sample size was based on logistical constraints, including laboratory capacity, available funding and participant availability. Therefore, future studies should aim to include a larger and more representative sample to enhance the generalisability of the findings.

## Supporting information

Liu et al. supplementary material Liu et al. supplementary material

## Footnotes

1 Results of the count approach is summarised in Appendix A, and they demonstrate similar findings that Label 12 and Label 08 are the top two labels that resonate consumers the most.

## Supplementary material

For supplementary material accompanying this paper, visit https://doi.org/10.1017/S1368980025100542.
