# Supplementary figures and images for: Identifying the best FDA-endorsed healthy label designs through best-worst scaling experiment
Source: Public Health Nutr. 2025 Jun 16;28(1):e104. doi: 10.1017/S1368980025100542 (PMC12264771; doi:10.1017/S1368980025100542)

**Appendix A**


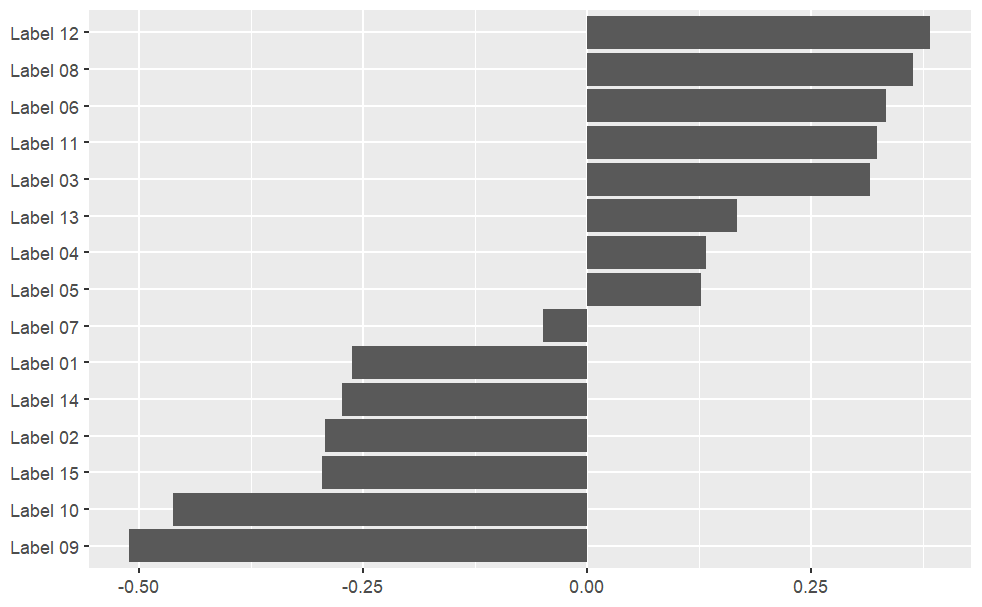


**Figure A1.** Preference orders of labels by count approach.

Supplement: Liu et al. supplementary material [file S1368980025100542sup001.docx]
